# Metformin increases glycolysis and the stress-induced cytokine GDF15 but not FGF21 in humans

**DOI:** 10.3389/fendo.2026.1797525

**Published:** 2026-03-18

**Authors:** Kristoffer J. Kolnes, Pauline M. Møller, Rikke Kruse, Mette Marie H. Christensen, Rasmus Kjøbsted, Martin Hey-Mogensen, Birgitte Andersen, Aase Handberg, Kurt Højlund

**Affiliations:** 1 Steno Diabetes Center Odense, Odense University Hospital, Odense, Denmark; 2 Department of Clinical Research, University of Southern Denmark, Odense, Denmark; 3 Department of Clinical Pharmacology, Odense University Hospital, Odense, Denmark; 4 August Krogh Section for Molecular Physiology, Department of Nutrition, Exercise and Sports, University of Copenhagen, Copenhagen, Denmark; 5 Global Drug Discovery, Novo Nordisk A/S, Måløv, Denmark; 6 Department of Clinical Biochemistry, Aalborg University Hospital, Aalborg, Denmark; 7 Department of Clinical Medicine, Aalborg University, Aalborg, Denmark

**Keywords:** FGF21, GDF15, glycolysis, metformin, mitochondrial respiration, intestine

## Abstract

**Background:**

Metformin lowers glucose by acting on the liver and the gastrointestinal tract and may reduce body weight by increasing circulating levels of the stress-induced cytokine GDF15. The tissue responsible for the release of GDF15 and whether this is paralleled by the induction of another, mainly liver derived, stress-responsive cytokine, FGF21, remains unclear.

**Objective:**

We examined the effect of metformin on GDF15 and FGF21 in humans and in intestinal cells *in vitro*.

**Methods:**

In a randomized, cross-over trial, 34 healthy individuals completed a 42-h fast twice, either with or without prior treatment with metformin for a week. Glucose metabolism was assessed using [3-^3^H]-glucose and indirect calorimetry and blood samples were drawn for the analysis of plasma metformin and serum GDF15 and FGF21. The effects of metformin on the expression and secretion of GDF15 and FGF21, and on mitochondrial respiration and glycolysis were examined in human intestinal epithelial cells (Caco-2).

**Results:**

Metformin increased glucose utilization (p=8.9x10^-13^) due to increased glycolysis (p=7.6x10^-13^) *in vivo*. This was accompanied by increased serum GDF15 (1004±61 vs 607±89 ng/ml; p<0.001), whereas serum FGF21 (146±30 vs 156±29 ng/ml; p=0.65) was unaltered. The change in serum GDF15 did not correlate with plasma metformin levels. *In vitro*, metformin markedly increased mRNA levels and secretion of GDF15, whereas FGF21 levels were not detectable in Caco-2 cells or media. Moreover, metformin dose-dependently inhibited mitochondrial respiration and increased glycolysis *in vitro*.

**Conclusions:**

The metformin-induced increase in serum GDF15, but not the liver-derived FGF21, in humans is consistent with the actions of metformin in human intestinal cells *in vitro*. These findings corroborate with recent studies demonstrating the gastrointestinal tract is an important site of metformin action.

**Clinical Trial Registration:**

ClinicalTrials.gov, Identifier NCT01400191.

## Introduction

Metformin is the most widely prescribed glucose-lowering drug worldwide in the management of type 2 diabetes (1). Although weight loss is often desired in patients with type 2 diabetes, metformin has shown little or no effect on body weight in this condition (2, 3). Nevertheless, metformin has been shown to reduce the risk of type 2 diabetes in individuals at high risk (2), an effect that has mainly been ascribed to a modest metformin-induced weight loss in individuals with prediabetes (2, 4). Despite extensive research on metformin, its mechanisms and sites of action are not fully understood (5). While suppression of the endogenous hepatic glucose production (EGP) has traditionally been thought to drive the glucose-lowering effect of metformin (6), there is now accumulating evidence suggesting that the gastrointestinal (GI) tract is a main contributor to the glucose-lowering effect of metformin in humans. Thus, the concentration of metformin in the intestine is 30–300 fold higher than that in the circulation (7) and markedly increases 18F-FDG uptake and glucose utilization in both the small intestine and colon in humans (8, 9). Furthermore, delayed-release metformin show the same glucose-lowering effect as the standard immediate-release formulation of metformin despite lower bioavailability (10), while intravenous administration of metformin reduces plasma glucose less than oral administration (11, 12). Additionally, several studies have recently demonstrated that metformin increases glucose utilization and hepatic glucose production, contrary to the proposed mechanism of action in the liver (13–15). Mechanistically, *in vitro* research consistently shows that metformin effectively inhibits mitochondrial respiration by targeting respiratory complex I in various tissues (16, 17) and, more recently, also in intestinal organoids (18), supporting its action in the intestines.

Growth differentiation factor 15 (GDF15) and fibroblast growth factor 21 (FGF21) are stress-induced cytokines secreted mainly in response to mitochondrial stress through activation of the integrated stress response (ISR) pathway (19, 20). GDF15 is induced in and secreted by most cell types in response to stress (21–26), whereas FGF21 is primarily expressed in and secreted by liver cells (27). GDF15 was recently suggested to mediate metformin-induced weight loss in mice through suppression of food intake via activation of GDNF family receptor α-like (GFRAL) in the hindbrain (28, 29). FGF21 serves as a pleiotropic molecule with a broad effect on glucose- and lipid metabolism and may even induce weight loss (30). Metformin increases circulating levels of GDF15 by ~1.5 fold in most studies of individuals with either type 2 diabetes, overweight/obesity, or prediabetes (29, 31–36). This increase in circulating GDF15 is believed to result from mitochondrial stress induced by the ability of metformin to inhibit respiratory complex I (37). However, the specific tissue responsible for metformin-induced GDF15 secretion remains under debate. *In vitro* studies have shown that metformin upregulates both GDF15 and FGF21 expression in hepatocytes (38), suggesting that if the liver is the primary target of metformin, elevated circulating levels of both stress-induced cytokines would be expected in humans. Although mitochondrial dysfunction induces both GDF15 and FGF21 through the ISR pathway (19, 39), the tissue responsible for the metformin-induced increase in plasma GDF15 and whether metformin also increases the secretion of FGF21 in humans remain to be investigated.

In the present study, we investigated the *in vivo* effect of metformin on serum GDF15 and FGF21 levels and their correlations with measures of glucose metabolism and plasma metformin during a glycogen-depleted state induced by a 42 h fast in healthy lean individuals. Subsequently, we conducted follow-up *in vitro* experiments using human intestinal epithelial Caco-2 cells to explore the dose-dependent effects of metformin on the expression and secretion of GDF15 and FGF21, as well as potential cellular and molecular mechanisms involved.

## Material and methods

### Human study

This is a secondary report of a randomized, cross-over trial of 34 healthy individuals (**Supplementary Table 1**). Baseline characteristics, and biochemical, and metabolic data have been reported previously (13). In brief, the participants were randomized to seven days of treatment with either placebo or metformin with a washout period of a minimum of four weeks before cross-over. The participants completed a 36 h fast at the end of each treatment period, prior to the assessment of glucose metabolism. Metformin doses were as follows; 500 mg twice the first day, 500 and 1000 mg the second day, and then 1000 mg twice daily for the preceding 4 days, concluding with 1000 mg in the morning immediately before starting examination, and hence after 36 h fast. Glucose metabolism was assessed using a primed-constant intravenous infusion of [3-^3^H]-glucose for six hours, after which the effects of the 42 h fast were evaluated as described. The fast was performed for 42 h to ensure that the main contribution to glucose production was hepatic gluconeogenesis, as liver glycogen will be depleted at this time (40). Blood samples for measurement of plasma metformin, free fatty acids (FFA) and glucagon, and serum insulin, C-peptide and cortisol were obtained every 30–60 min, whereas blood samples for plasma glucose, lactate, and [3-^3^H]-glucose were obtained every 15 min during the 6 h infusion period. All the above measurements were analyzed as described previously (13). Danish Health and Medicine Authorities, the Regional Ethical Committee of Southern Denmark and the Danish Data protecting Agency, approved the study. All participants gave written informed consent to participate. The study was carried out in accordance with the Helsinki declaration.

### Indirect calorimetry

ParvoMedics TrueOne 2400 (Sandy, UT, USA) automated gas analysis system was used to perform indirect calorimetry. The average gas exchange rates recorded over the two 30-min steady-state periods (90–120 min and 330–360 min) after an equilibration period of 10 min, were used to calculate rates of glucose oxidation and lipid oxidation as well as respiratory exchange ratio (RER) and resting energy expenditure (REE).

### Tracer determined glucose turnover

After a 2 h basal tracer equilibration period, the glucose turnover rates for the period from 2–6 h were calculated as described previously (13). The rates of glucose appearance (Ra) and glucose disposal (Rd) were calculated using Steele’s non-steady-state equations as described (41). Ra was assumed to be equal to EGP during the last 4 h. The rates of glycolytic flux were calculated as described in detail previously (42). The difference between Rd and glucose oxidation was used to calculate non-oxidative glucose metabolism (NOGM).

### Assays for serum GDF15 and FGF21

GDF15 was measured in duplicates on fasting serum samples by the human GDF-15 Quantakine ^®^ ELISA assay essentially as described (R&D Systems, Abingdon, UK). Mean coefficient of variation on duplicates was 3.6%. Internal controls were run in two levels and between run coefficient of variation were 7.4% (121.4 pg/mL) and 1.7% (686 pg/mL), three runs. FGF21 was measured on fasting serum samples by the human FGF21 Quantikine^®^ ELISA assay essentially as described (R&D Systems, Abingdon, UK). Internal controls in three levels were run in duplicates. The coefficient of variation was 0.4% (223 pg/mL), 0.2% (632 pg/mL) and 3.1% (1105 pg/mL). Serum GDF15 and FGF21 were measured at the end of the examination periods.

### Cell culture

The human intestinal epithelial cell line, Caco-2, was obtained from the American Type Culture Collection (ATCC) and cultured under standard conditions (37 °C, 5% CO_2_). Caco-2 cell line is an immortalized human colon cancer cell line that spontaneously differentiate in culture to form a heterogeneous mix of intestinal cells. The cells were maintained in high glucose Dulbecco’s Modified Eagle Medium (DMEM) GlutaMax (Gibco, CA, US) supplemented with 10% Fetal Bovine Serum (FBS) (Gibco, CA, US), 1% penicillin-streptomycin (P/S) solution (Gibco, CA, US) and 1x MEM Non-Essential Amino Acid (NEAA) Solution (Gibco, CA, US). The cells used for the secretion assays and transcriptional analyses were seeded into 24-well plates and allowed to spontaneously differentiate for 22 days. Subsequently, the cells were stimulated with either 0.3 mM, 1 mM, or 3 mM metformin solution (Merck, NJ, US) for 6 or 22 h in DMEM, no glucose media (Gibco, CA, US) supplemented with either 5.5 mM, 11 mM or 25 mM glucose, as well as 10% FBS (Gibco, CA, US), 1% P/S (Gibco, CA, US), 1x MEM NEAA (Gibco, CA, US) and 1 mM pyruvate (Gibco, CA, US). Controls were left untreated and maintained for 6 or 22 h in DMEM (Gibco, CA, US) with added glucose concentrations of 5.5 mM, 11 mM, or 25 mM. Prior to snap freezing, media was collected for the secretion assays, and the cells were briefly washed in phosphate buffered saline (PBS). For transcriptional analyses, the cells were analyzed in Beckman Coulter Lysis LBE buffer (Fisher Scientific, MA, US) with Beckman Coulter Proteinase K (Fisher Scientific, MA, US) and transferred to a deep well plate for RNA extraction. The cells used for measuring mitochondrial oxygen consumption rates (OCR) and extracellular acidification rates (ECAR) were seeded in a 96-well Seahorse Cell Culture Plate with a cell density of 7500 cells/well and differentiated for 22 days in DMEM GlutaMax (Gibco, CA, US) supplemented with 10% FBS (Gibco, CA, US), 1% P/S (Gibco, CA, US) and 1x MEM NEAA (Gibco, CA, US).

### Real-time quantitative PCR

RNA extraction from Caco-2 cells was performed on a Biomek i7 automated workstation (Ramcon, DK) using Beckman Coulter beads-based technology (Fisher Scientific, MA, US), according to the manufacturer’s instructions. The RNA was treated with RNase-Free DNase (Qiagen, Germany) and eluted in RNA-free water. Reverse transcription was performed from 500 ng of RNA using the iScript cDNA Synthesis Kit (Bio-Rad, CA, US). Real-time qPCR was performed on a Quantstudio 7 Flex Real-Time qPCR System (Applied Biosystems, MA, US) using TaqMan Fast Advanced Master Mix (Applied Biosystems, MA, US) as instructed by the manufacturer. The specific primer-probe pairs used were *GDF15* (Hs00171132_m1, Life-Technologies, CA USA), *FGF21* (Hs00173927_m1, Life-Technologies, CA USA) *SLC2A1* (Hs00892681_m1, Life-Technologies, CA, USA), *ATF4* (Hs00909569_g1, Life-Technologies, CA, USA), *DDIT3* (Hs00358796_g1, Life-Technologies, CA USA), and *GAPDH* (Hs02786624_g1, Life-technologies, CA, USA). Cycle values obtained from each transcript of interest were normalized to the reference gene *GAPDH*, which was stable under all conditions and then expressed relatively as 2^-ΔΔCt^ for the given primer. CT-values around 35 were considered the detection limit of the assay.

### Secretion assays

GDF15 and FGF21 secretion were measured in the media collected from the Caco-2 cells used for transcriptional analyses. The protein concentrations of GDF15 and FGF21 were measured in media collected after 22 h of metformin treatment at concentrations of 0.3 mM, 1 mM, or 3 mM in media containing either 5.5 mM, 11 mM, or 25 mM glucose. Media stored at -80 °C was thawed on ice, and GDF15 and FGF21 protein levels were measured using the Human GDF-15 Quantikine ELISA Kit (R&D Systems, MN, US) and Human FGF-21 Quantikine ELISA Kit (R&D Systems, MN, US) according to the manufacturer’s protocol. The optical density was measured on a Spectramax Plus 384 Microplate Reader (Molecular Devices, CA, USA) at wavelengths of 450 nm and 570 nm.

### Seahorse bioanalyzer assay

Mitochondrial respiration (OCR) and ECAR were measured on a Seahorse XFe96 Analyzer (Agilent, CA, US) using the Seahorse XF Cell Mito Stress Test (Agilent, CA, US). The Mito Stress assay was performed according to the manufacturer’s instructions. Briefly, OCR and ECAR were measured in Caco-2 cells in response to acute metformin stimulation 20 min before the first measuring time point and in response to chronic stimulation with pre-treatment of metformin for 24 hours prior to the first measuring time point. For both the acute and chronic experiment, the outer wells of the 96-well plate were used as vehicles in addition to at least 12 controls for either oligomycin or carbonyl cyanide 4-(trifluoromethoxy)phenylhydrazone (FCCP) treatment. The cells were treated with metformin solutions (Merck, NJ, US) in concentrations of 0.3 mM, 1 mM, or 3 mM. The cartridge was hydrated with 200 µL sterile water in a non-CO_2_ incubator for 16–20 h before the experiment and replaced with 200 µL of XF calibrant. The cells were kept in Seahorse XF DMEM assay media (Agilent, CA, US) supplemented with 10 mM glucose, 1 mM NaPyruvate, and 2 mM glutamine during the measurements. Both experiments were performed at 37 °C, and the mitochondrial inhibitors were added in the following concentrations: Oligomycin (2 µM), FCCP (1µM), and Rotenone and Antimycin A (0.5 µM).

### Statistics

Statistical analyses of *in vivo* data were performed using STATA 16.0 (StataCorp, TX, USA). Paired student’s *t*-tests were used to determine significant differences between treatment with either placebo or metformin. The relationships between selected variables were examined by calculating Pearson’s correlation coefficients. Statistical analyses of *in vitro* data were performed using GraphPad Prism 9.0. Statistical testing was performed between stimulated cells and controls and between non-treated cells at different glucose concentrations using two-way ANOVA analyses followed by Tukey’s *post-hoc* testing unless otherwise stated. All data are presented as means ± SEM or SD. P-values below p<0.05 were considered significant.

## Results

### Clinical and metabolic characteristics

The clinical and metabolic characteristics (**Supplementary Table 2**) have been reported previously (13). In brief, plasma glucose levels declined during the 6 h examination period after both placebo and metformin treatment, but did not differ significantly between treatment groups. However, metformin treatment for a week, with the last dose (1000 mg) given at the initiation of the examination, markedly increased both Rd (68.1 ± 1.1 vs 55.2 ± 0.9 mg/min/m^2^; p=8.9x10^-13^) and EGP (67.2 ± 1.2 vs 54.0 ± 1.0 mg/min/m^2^; p=7.6x10^-13^) after the glycogen-depleting fast (**Supplementary Table 2**). The increased glucose utilization was explained by increased rates of glycolytic flux (57.2 ± 1.1 vs 43.9 ± 0.9 mg/min/m^2^; p=1.7x10^-11^) and NOGM (64.4 ± 2.2 vs 49.6 ± 3.7 mg/min/m^2^; p=0.0024) after treatment with metformin and was consistent with increased plasma lactate levels (0.97 ± 0.03 vs 0.89 ± 0.03 mmol/l; p=0.002). Glucose and lipid oxidation, as well as RER and REE, were unaltered in response to metformin. Metformin treatment was accompanied by increased levels of serum cortisol and plasma glucagon (both p<0.05), which likely contributed to the counterregulatory increase in EGP.

### Serum GDF15 and FGF21 levels and correlation analyses

**Figure 1:**
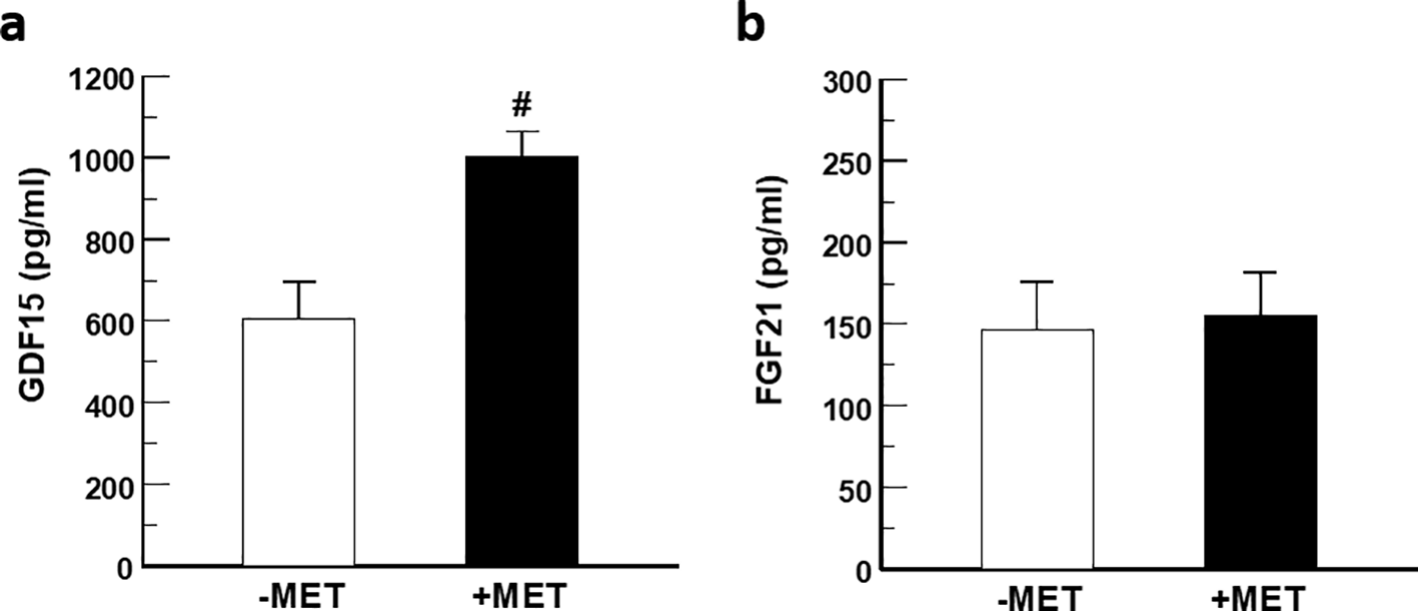
Effect of metformin on circulating GDF15 and FGF21. **(a)** Serum GDF15 **(b)** and serum FGF21 in healthy individuals fasted for 42 h without (white) and with (black) prior metformin (MET) treatment for seven days. Data is given in mean +/- SEM. #p<0.0001.

Treatment with metformin increased serum levels of GDF15 by 1.7 fold (1.004 ± 61 vs. 607 ± 89 ng/mL; p<0.001) after 42 h fast (**Figure 1a**). Conversely, no significant changes in serum FGF21 levels were observed (156 ± 29 vs. 146 ± 30 ng/mL; p=0.65) in response to metformin (**Figure 1b**).

Correlation analyses (**Supplementary Table 3**) were performed to examine the potential relationship between clinical and metabolic characteristics and circulating levels of GDF15 and FGF21 at baseline (without metformin). At baseline (n=34), serum GDF15 correlated inversely with height (*r* = -0.47, p=0.005), weight (*r* = -0.495, p=0.003), and plasma glucose (*r* = -0.478, p=0.004), but not significantly with BMI (*r* = -0.28, p=0.10), age, or plasma FFA or lactate (all p<0.76). At baseline (without metformin), serum GDF15 was negatively associated with EGP (*r* = -0.39, p=0.021) and also tended to correlate inversely with Rd (*r* = -0.33, p=0.056), whereas no associations were found between serum GDF15 and glycolytic flux, NOGM, substrate oxidation, or REE during the last 30 min of the examination. Baseline serum FGF21 tended to correlate positively with plasma FFA (*r* = -0.338, p=0.051) and negatively with plasma glucose (*r* = -0.332, p=0.056) but was not associated with age, weight, height, or BMI. Baseline serum FGF21 did not correlate with either Rd, EGP, NOGM, substrate oxidation or REE. However, serum FGF21 correlated with glycolytic flux (*r* = 0.34, p=0.049).

**Figure 2:**
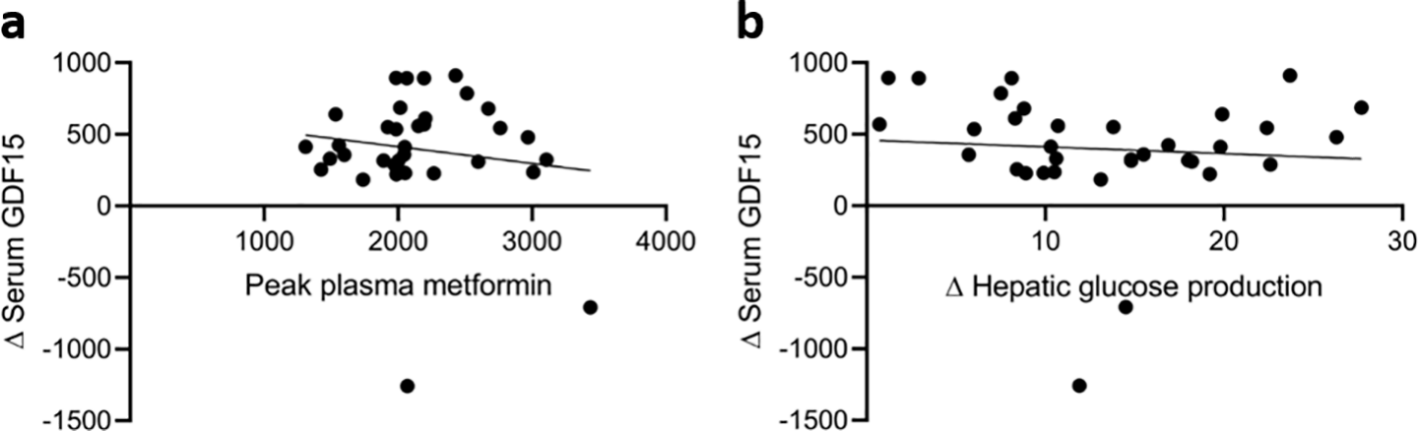
The relationship between serum GDF15, plasma metformin and hepatic glucose production. **(a)** Scatter plots of the associations between changes (Δ) in serum GDF15 and peak plasma metformin and **(b)** changes (Δ) in hepatic glucose production.

To explore possible mechanisms related to the effect of metformin on GDF15, we examined the correlation between the metformin-induced (metformin vs. placebo) increase in serum GDF15 and observed changes in measures of glucose metabolism and plasma metformin (**Supplementary Table 3**). The increase (Δ-value) in serum GDF15 caused by metformin treatment did not correlate with the maximal concentration (p=0.42) or area under the curve (AUC) of plasma metformin (p=0.41) (**Figures 2a, b**). Furthermore, the metformin-induced increase in GDF15 did not significantly correlate with any of the observed increases in Rd (p=0.71), EGP (p=0.66), or glycolytic flux (p=0.84).

### Effect of metformin on the expression of GDF15, FGF21, and related genes *in vitro*

**Figure 3:**
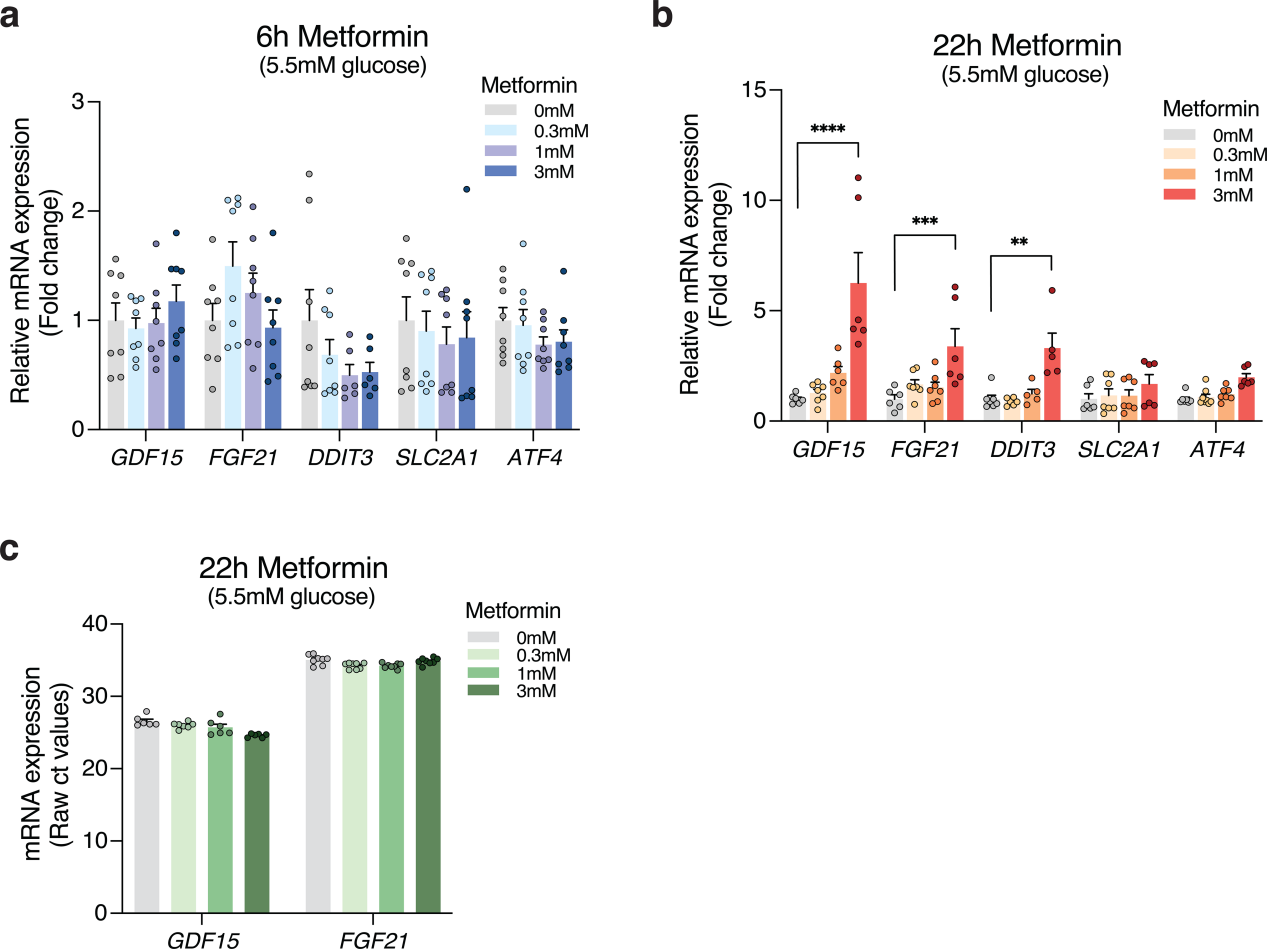
Metformin increases mRNA levels of *GDF15* in human intestinal Caco-2 cells with no related increase in FGF21. Relative mRNA expression of *GDF15, FGF21, SLC2A1*, and the ISR genes *ATF4* and *DDIT3* in differentiated Caco-2 cells after **(a)** 6 h or **(b)** 22 h without treatment (0 mM) or 0.3 mM, 1 mM, or 3 mM metformin treatment at media glucose concentrations of 5.5 mM **(c)** Raw ct values of *GDF15* and *FGF21* at 5.5 mM glucose. n = 6-8, 2 wells from 4 independent experiments evaluated in parallel. The data is presented as mean +/- SEM. **p<0.01, ***p<0.001, and ****p<0.0001 as indicated.

To investigate the effect of metformin on the expression of *GDF15*, *FGF21*, *SLC2A1* (encoding GLUT1), and the ISR-related genes *ATF4* and *DDIT3* (encoding the transcription factor CHOP), Caco-2 cells were treated with increasing metformin concentrations (0.3 mM, 1 mM, or 3 mM) or without metformin as control (vehicle). The cells were incubated with metformin or vehicle for 6 or 22 h in media with increasing glucose concentrations of either 5.5 mM, 11 mM, or 25 mM. In vehicle-treated cells, transcript levels of *GDF15*, *FGF21*, *SLC2A1, ATF4*, and *DDIT3* remained constant independently of glucose concentrations and time points (e.g., 6 h and 22 h) (**Supplementary Figures 1a, b**). Likewise, after 6 h of metformin stimulation, the mRNA levels of these genes showed no significant changes at any glucose concentrations (**Figure 3a**; **Supplementary Figures 2a, b**). However, after 22 h treatment with 3 mM metformin, a robust increase in mRNA expression was observed for several genes (**Figure 3b**; **Supplementary Figures 2c, d**). These included a 6-fold increase in *GDF15* levels at 5.5 mM glucose (p<0.0001) with similar effects observed at other glucose concentrations (i.e., 11 mM: ~3.5-fold, 25 mM: ~4.5-fold), as well as elevated expression of the ISR gene *DDIT3* by 2–3 fold at all examined glucose concentrations (all p<0.05), while *ATF4* and *SCL2A1* expression were unaffected by metformin at all glucose concentrations (**Supplementary Figures 2c, d**). *FGF21* mRNA levels increased following 22 h of 3 mM metformin treatment when compared to vehicle at all glucose concentrations (**Figure 3b**; **Supplementary Figures 2c, d**). However, given the high ct values of *FGF21* (ct: ~33–36) compared to *GDF15* ct values (ct: ~4–28), these findings rather indicate that FGF21 expression is barely detectable in Caco-2 cells (**Figure 3c**).

### Effect of metformin on the secretion of GDF15 and FGF21 *in vitro*

**Figure 4:**
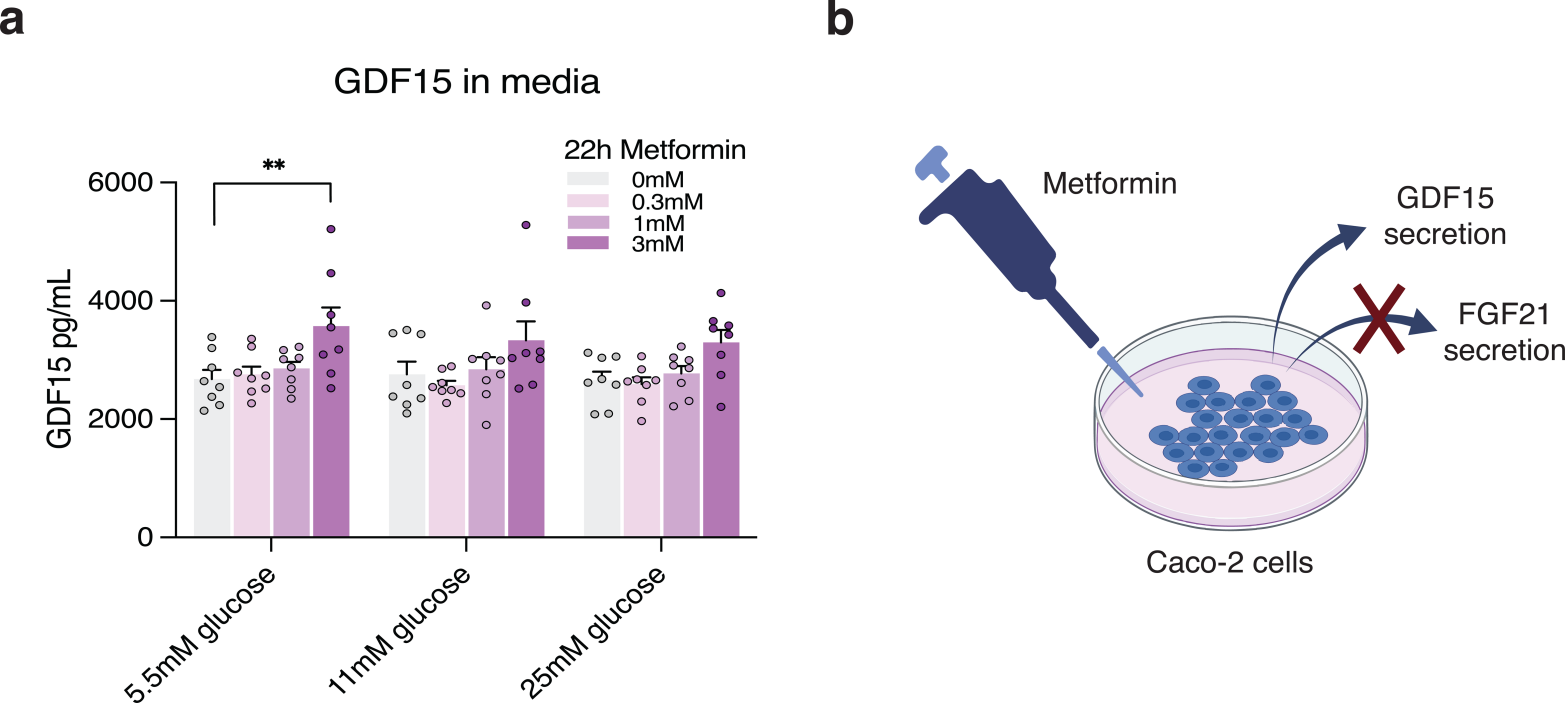
GDF15 secretion is increased in Caco-2 cells upon chronic metformin treatment. **(a)** GDF15 concentration in media collected from non-treated (0 mM) Caco-2 cells kept in media with glucose concentrations of 5.5 mM, 11 mM, or 25 mM or from cells treated with 0.3 mM, 1 mM, or 3 mM metformin at similar glucose concentrations. **(b)** A schematic representation of metformin-stimulated GDF15 secretion from Caco-2 cells, where FGF21 protein levels were undetectable. n = 8, 2 wells from 4 independent experiments evaluated in parallel. The data is presented as mean +/- SEM. **p<0.01, determined by two-way ANOVA analysis with Dunnett correction. **Figure 3b** is created in BioRender. Møller, P. (2025) https://BioRender.com/o87c709.

To support our transcript data, we evaluated GDF15 and FGF21 secretion by measuring protein concentrations in media collected from Caco-2 cells following 22 h of metformin stimulation (**Figure 4a**). Consistent with our gene expression data, 3 mM metformin significantly increased GDF15 protein levels in media at 5.5 mM glucose from ~2700 pg/mL to ~3670 pg/mL (p=0.0046) compared to vehicle, while no effect was observed at lower metformin concentrations. This effect tended to be maintained at higher glucose concentrations, though to a lesser extent, with an increase of ~3300 pg/mL GDF15 upon 3 mM metformin treatment at both 11 mM (p=0.113) and 25 mM (p=0.066) glucose compared to vehicle. In agreement with the low expression levels, we were unable to detect FGF21 in media collected from vehicle-treated or metformin-treated Caco-2 cells maintained in media with either 5.5 mM, 11 mM, or 25 mM glucose (data not shown) (**Figure 4b**).

### Effect of metformin on mitochondrial respiration *in vitro*

**Figure 5:**
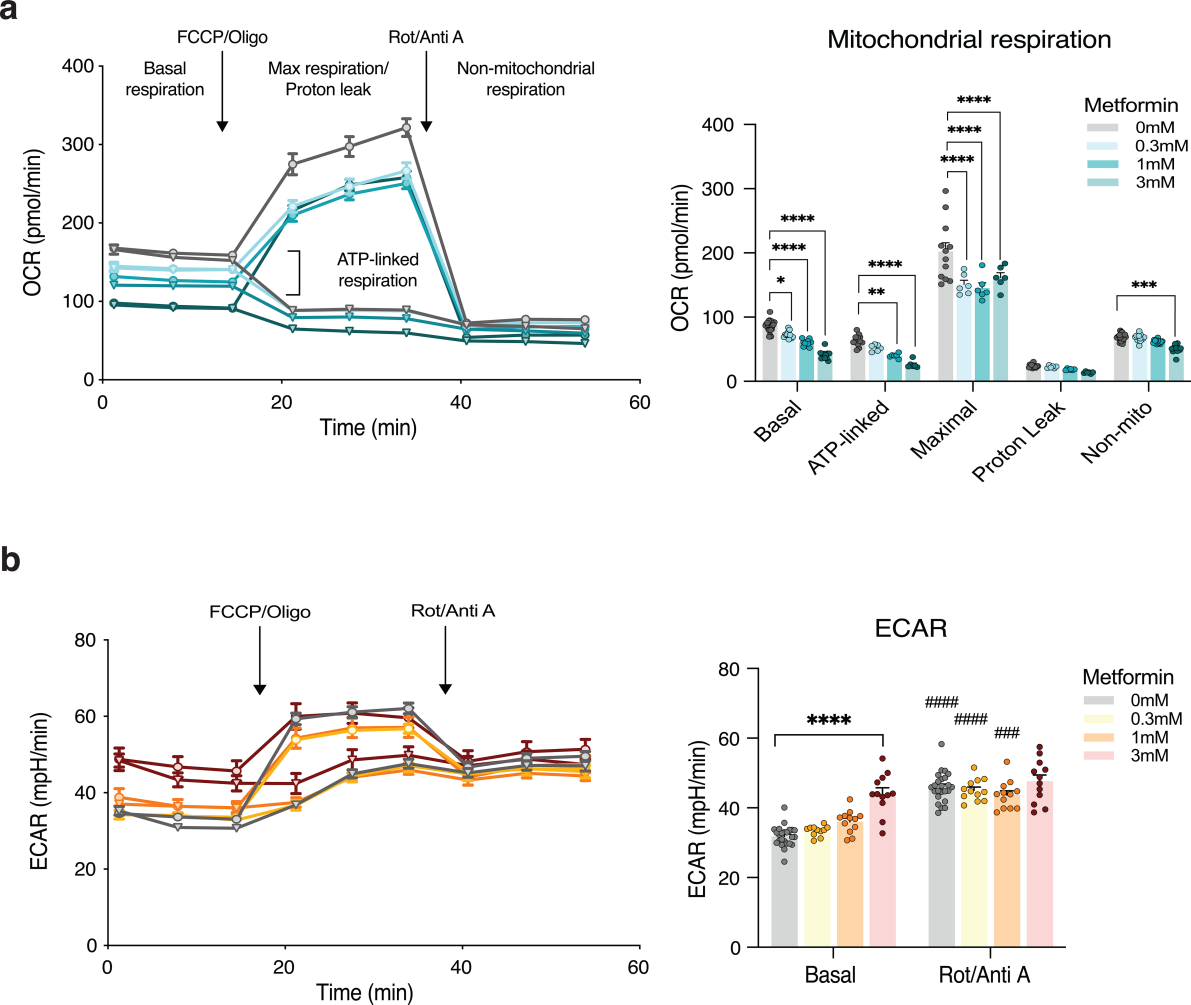
Metformin strongly inhibits mitochondrial respiration in intestinal cells with concomitant induction of the extracellular acidification rate. **(a)** Changes in oxygen consumption rate (OCR) and **(b)** extracellular acidification rate (ECAR) during a mitochondrial stress test of human intestinal Caco-2 cells pre-treated for 24 h with 0.3 mM, 1 mM or 3 mM metformin or left untreated (0 mM). Arrows indicate the timepoint for when the mitochondrial inhibitors were added; Oligo (Oligomycin) at 2 µM, FCCP (Carbonyl cyanide-4 (trifluoromethoxy) phenylhydrazone) at 1 µM, and Rot/Anti A (Rotenone, and Antimycin A) at 0.5 µM. n=24 wells for baseline and Rot/Anti A measurements of OCR (pmol/min) and ECAR (mph/min) in controls. n=12 for OCR and ECAR at baseline or after Rot/Anti injection in metformin-treated wells (i.e., 0.3 mM, 1 mM, and 3 mM). Half of the plate was treated with either oligo or FCCP, (n=12 for vehicle and n=6 for metformin-treated cells). The data are presented as mean +/- SEM. *p<0.05, **p<0.01,***p<0.001, and ****p<0.0001; ###p<0.001 and ####p<0.0001 vs basal using same metformin concentrations.

To delineate the effects of metformin on mitochondrial respiration in human intestinal cells *in vitro*, we measured OCR and ECAR during a mitochondrial stress test in Caco-2 cells treated with 0.3 mM, 1 mM, or 3 mM metformin either acutely or chronically for 24 h. The OCR and ECAR remained unaltered in response to acute metformin stimulation both at baseline and following treatment with mitochondrial uncouplers and inhibitors (i.e., FCCP, oligomycin, and Rotenone/Antimycin A) (**Supplementary Figure 3**). Conversely, 24 h pretreatment with either 0.3 mM, 1 mM, or 3 mM metformin induced a strong dose-dependent reduction in baseline OCR compared to controls (**Figure 5a**). Metformin also decreased ATP-linked- and maximal mitochondrial respiration, while non-mitochondrial respiration was only decreased following 3 mM metformin and no change was observed in proton leak associated OCR. Baseline ECAR increased by ~38% in Caco-2 cells following 24 h pretreatment with 3 mM metformin, while no effect was observed at lower concentrations (**Figure 5b**). The production of protons through glycolysis and lactate (i.e. from glycolytic pyruvate) can be used as a measure of increased glycolytic flux and is commonly estimated by the ECAR (43). However, as increased flux through the tricarboxylic acid cycle also contributes to increased ECAR (i.e., through CO_2_ buffering), the glycolytic flux should only be estimated during a Mito Stress Assay after complete mitochondrial block (i.e., following rotenone/antimycin A), where an increase or unaltered ECAR indicate glycolytic acidification (43). Here, we discovered that when treating Caco-2 cells with 3 mM metformin the increase in ECAR following complete mitochondrial block (i.e., Rot/Anti A) was completely blunted compared to controls, thus indicating that the metformin-induced increase in baseline ECAR is mainly contributed by increased glycolytic flux (**Figure 5b**).

## Discussion

The principal finding in our study was that metformin increased serum GDF15 in young, healthy individuals who fasted for 42 hours without a concomitant increase in serum FGF21. The metformin-mediated increase in serum GDF15 was accompanied by increased glucose utilization due to enhanced glycolysis as well as increased EGP, which probably prevented a further decline in glucose levels, as reported (13). The lack of increase in serum of the liver-derived FGF21 together with the absent correlation between the increase in serum GDF15 and peak plasma metformin concentrations, argue against the liver as the main site of metformin action, and in agreement with existing data suggest the intestine as a possible main site of metformin action *in vivo* in humans. We, therefore, decided to validate our findings in a human intestinal epithelial cell line, and investigate potential cellular and molecular mechanisms involved. In line with our *in vivo* data, metformin increased the transcription and secretion of GDF15 in Caco-2 cells, whereas FGF21 secretion and transcription were barely detectable. This increase in GDF15 was accompanied by inhibition of mitochondrial respiration and increased glycolysis *in vitro*. Together, these observations are consistent with prior evidence indicating that metformin’s glucose-lowering action and its induction of GDF15 could primarily take place in the intestine.

The clinical part of our study extends previous cross-sectional (33, 34) and prospective (29, 31, 32, 35, 36) studies in humans, showing that treatment with metformin is associated with an increase in circulating levels of GDF15. In these studies, the participants had either type 2 diabetes (32–34), overweight/obesity, or prediabetes (29, 31, 35, 36), all conditions in which circulating levels of GDF15 are often elevated (29, 31–36). In most of these studies, serum GDF15 was increased by 1.4-1.5 fold in response to metformin, but up to 2.5 fold increases have been reported in shorter-term (<8 weeks) studies (29, 32). Here, we extend these findings to healthy lean individuals fasted for 42 hours and in the glycogen-depleted state, showing that treatment with metformin for a week increases serum GDF15 by 1.7-fold. This increase was observed in addition to the approximately 80% rise in serum GDF15 reported following 48 hours of fasting in humans (44). Interestingly, the metformin-induced increase in serum GDF15 was paralleled by increased glucose utilization explained by a 30% increase in glycolysis. To our knowledge, these findings for the first time suggest a coupling between the ability of metformin to increase both circulating GDF15 and glycolysis in humans. As expected, the metformin-induced increase in glycolysis was accompanied by a small increase in plasma lactate (13), which might be even higher if measured in the portal vein as shown recently (45).

Several studies in humans have shown that the site of action of metformin, including its ability to induce endogenous GDF15, could be the GI tract. Clinically, this is supported both indirectly (9) and directly (8) by FDG-PET imaging studies showing that metformin increases 18F-FDG bowel uptake and increases glucose uptake/glucose utilization 2-3-fold in both in the colon and small intestine from the basolateral site (46). In addition, studies of delayed-released metformin show similar glucose-lowering effects as standard (immediate-release) metformin in patients with type 2 diabetes despite reduced systemic bioavailability (10), and the ability of metformin to reduce glucose levels is lower with intravenous than oral administration in humans (11, 12). Consistent with these findings, we found no correlation between peak plasma levels of metformin and the increase in serum GDF15 levels supporting a local action in the intestine.

Recent studies have shown that metformin primarily induces GDF15 in the small intestine, colon, and kidney in high-fat diet (HFD) mice (29), and that the metformin-induced increase in the expression and release of GDF15 from the small intestine is mediated by inhibition of mitochondrial respiration resulting in increased glycolysis and hence glucose utilization in mice (17, 18). Although our study was not designed to prove this in humans, our results provide evidence that treatment with metformin increases glycolysis, which could potentially occur via inhibition of mitochondrial respiration, and that this is accompanied by elevated serum GDF15 levels. The accompanying increase in EGP in response to metformin suggests that these effects of metformin are not mediated through actions in the liver. Our findings are supported by a recent study showing that a single dose of metformin increases intestinal glycolysis and lactate release in patients with an intrahepatic portosystemic stent (47). The observed lack of correlation between the metformin-induced increases in serum GDF15 and glycolysis in our study suggests that other tissues, such as the kidney, may contribute to the increase in serum GDF15 (48), which might be independent of a rise in glycolysis.

As reported previously, the metformin-induced increase in glucose utilization was fully counteracted by an increased EGP from gluconeogenesis (13), and this likely protected against hypoglycemia in this fasting situation. Our original findings were recently confirmed by other groups, including in patients with type 2 diabetes (14, 15). Thus, our results show that neither metformin nor the 1.7-fold increase in serum GDF15 reduces EGP in healthy individuals fasted for 42 h. Treatment with recombinant GDF15 in pharmacological doses was recently reported to improve insulin action on EGP in the liver via GFRAL-β-adrenergic dependent signaling mechanisms (49) and a feedback to the liver in mice (46). Our data demonstrate that if metformin directly or via the induction of endogenous GDF15 has any inhibitory effect on EGP via activation of the GFRAL receptor in the brain, it is completely overruled by the glucose counterregulatory response driven by the increased glucose utilization (glycolysis). This makes metformin a safe drug regarding the risk of hypoglycemia. Another observation was that the 70% induction of GDF15 was not accompanied by increased plasma FFA levels, fatty acid oxidation, or energy expenditure in humans. This is in contrast to studies in mice, which have shown that high doses of GDF15 via the GFRAL–β-adrenergic dependent signaling axis increases fatty acid oxidation (50–53) and energy expenditure (52), suggesting that this response may not be translated into humans or that much higher levels of GDF15 than those induced by metformin are needed to elicit these responses.

A major novel finding of this study is the fact that while metformin markedly increased serum GDF15, it did not increase serum FGF21 in healthy young individuals fasted for 42 h. FGF21 is similar to GDF15, a stress-responsive mitokine, which shows increased expression and secretion in response to inhibition of mitochondrial respiration due to activation of ISR pathways (20, 39). Thus, if mitochondrial respiration was inhibited in a tissue known to be a major site of expressing and releasing FGF21, the liver, it would be expected that both circulating levels of GDF15 and FGF21 would increase in response to metformin treatment, at least if the circulating concentrations of metformin were sufficiently high to elicit these responses. In support of this hypothesis, parallel increases in GDF15 and FGF21 levels are seen in both mitochondrial myopathies and hepatopathies in humans (54–57). Moreover, the stress induced by acute exercise increases the circulating levels of both GDF15 and FGF21 in humans (58–61), and in this situation, these stress-induced cytokines seem to be released from the liver (61, 62). While GDF15 is ubiquitously expressed (21–26), FGF21 is, to a major extent, expressed in the liver as well as the pancreas and testis (27). Therefore, our findings add further support to recent observations that the metformin-mediated induction of GDF15 levels are taking place in the GI tract or kidneys and not in the liver (29), and, thus, lend support to studies showing that metformin increases GDF15 and glycolysis through its action in the GI tract (46).

As our experimental set-up *in vivo* included fasting for 36 hours before the final dose of metformin and the subsequent 6-h examination, carbohydrates from food in the gut could not be responsible for the increased glycolysis observed in response to metformin in our study. Interestingly, recent FDG-PET studies have shown that metformin promotes glucose flux from the circulation into the intestine explaining a source of glucose fueling for intestinal glycolysis (63, 64). This suggest that the required enhanced glucose availability could be explained by the observed increase in EGP under these conditions. Another finding in these FDG-PET studies was that metformin also promotes excretion of glucose into the intestinal lumen (63, 64), where it is digested by gut microbiota to increase the content of short chain fatty acids. This emphasizes that metformin-induced changes in the gut microbiome may contribute to its metabolic effects including improved glucose homestasis (65).

Indeed, metformin in high concentrations can induce FGF21 expression and secretion in human hepatocytes *in vitro* (38), but the plasma concentrations of metformin reaching the liver in humans are probably too low to induce FGF21 *in vivo*. Fasting for seven days but not for two-three days (66, 67) has been shown to increase circulating FGF21, with a diurnal pattern in humans (66). However, other known stimulators of FGF21, such as glucose and FFA levels (68–70), did not show different responses to the 42 h fast between the two groups in our study, excluding changes in these substrates as the reason for the lack of changes in FGF21 in response to metformin. Indeed, other groups have examined the effect of metformin on plasma FGF21 in humans with different findings. Thus, in a small cohort of patients with type 2 diabetes, treatment with metformin for 6 months increased serum FGF21 (20). However, in a much larger cohort of patients with type 2 diabetes, both plasma FGF21 and hsCRP were markedly reduced after treatment with metformin for 12 weeks (71). As circulating FGF21 levels are often elevated in patients with type 2 diabetes (71), the latter study suggests that metformin may reduce FGF21 by alleviating systemic low-grade inflammation.

Given that our findings demonstrate that metformin increases serum GDF15 and whole-body glycolysis but not serum FGF21, we examined if the ability of metformin to induce GDF15 but not FGF21 could be validated in human intestinal epithelial cells *in vitro* and explored the cellular and molecular mechanism involved. Consistent with previous preclinical studies in the intestine of mice, intestinal organoids, and human intestinal cells (18, 29, 48, 72), we demonstrate that the gene expression and secretion of GDF15 are markedly induced by treatment with a high dose of metformin for 22 hours in human intestinal Caco2-cells, which is in line with recent reports (29, 44). This effect was associated with transcriptional activation of *DDIT3*, encoding the protein CHOP, which is a critical factor in activation of the ISR pathway (44, 73). The absent effect seen after 6 hours and at lower doses of metformin may suggest that a certain amount of time and metformin are needed for metformin to accumulate in sufficiently high concentrations in the Caco2-cells to achieve these effects. These findings support the notion that metformin concentrations are 30–300 times higher in the gut than in the circulation *in vivo* (7) and also suggest that the abundance of different known metformin transporters in the intestine could play a role in metformins excessive accumulation in the GI tract (74), hence providing the explanation for this tissue being a primary site of action for metformin.

In our *in vitro* experiments, we exposed the Caco2-cells to high concentrations of metformin (3 mM), and it could be questioned to what extent these concentrations relate to luminal versus intracellular intestinal concentrations in humans, and whether the observed effects on mitochondrial respiration and GDF15 expression are likely to reflect physiological conditions. However, assuming an up to 300 times higher concentration of metformin in the gut than in the circulation in humans *in vivo* (7), the previously reported plasma concentrations of metformin at the time the samples were taken (~1200 ng/ml corresponding to 9.6 μmol/l) in our study (75) would equal 2.9 mM in the intestine. This suggest that the observed effects of metformin 3 mM in Caco2 cells may well reflect what happens intracellularly in human intestinal cells *in vivo*. However, we cannot exclude that metformin also accumulates in specifically the Caco2 cells after 2 h exposure leading to even higher concentrations than 3 mM, and, therefore, that the observed inhibition of mitochondrial respiration and increased expression and secretion of GDF15 reflect even higher concentrations.

Metformin has been shown to cause mitochondrial stress e.g., inhibition of complex I respiration in several cell types (16, 17). A recent study demonstrated that this included intestinal organoids and cells, in which the reduction in mitochondrial respiration was accompanied by increased glycolysis in response to metformin (18), which have also been shown in ileum and colon of animals on a HFD (46). In our study, the mitochondrial stress test revealed no effect on OCR or ECAR after acute stimulation with metformin for 20 min. This again suggests that more time is needed for metformin to accumulate to concentrations that can elicit its effects intracellularly. Metformin treatment markedly reduced mitochondrial respiration in a dose-dependent manner after 24 h, whereas only treatment with 3 mM metformin significantly increased glycolysis in the human intestinal Caco-2 cells. This is consistent with recent findings (18, 46) and these results support our clinical findings that metformin treatment increases whole-body glucose utilization due to increased glycolysis and suggests that this likely takes place in the intestine. However, in contrast to findings from a study in intestinal organoids reporting metformin-induced upregulation of *ATF4*, *GLUT1*, and glycolytic gene expression (18), we were unable to confirm an increase in *ATF4* or *GLUT1* expression in our experimental model. These results suggest that the observed enhancement in glycolytic flux may be mediated by alternative regulatory mechanisms. Accordingly, further investigations are warranted to elucidate the molecular pathways underlying the increase in glycolytic flux.

Intriguingly, metformin has been shown to reduce ROS production in various cell types (76, 77). Since increased ROS induced by hypoxia and mitochondrial stress has been shown to activate the ISR pathway via PERK/eIF2A/ATF4 signaling (78), this suggest the possibility that the reported metformin-induced activation of the ISR pathway, which is associated with metformins inhibitory effect on mitochondrial respiration (18, 19, 46), may be partially counteracted by a concomitant reduction in ROS production. These potential dual and opposing effects of metformin on the ISR pathway could explain the absent increases in mRNA levels of *ATF4* and *GLUT1* in response to metformin in our study.

Despite transcriptional activation of the ISR-pathway and the gene expression and secretion of the stress-induced cytokine GDF15, we found no clearly detectable mRNA levels or secretion of the other stress-induced cytokine FGF21 after a high dose of metformin for 22 hours in the human intestinal Caco-2 cells. To our knowledge, this has not been shown previously and these findings *in vitro* support our clinical findings that metformin treatment only increases circulating GDF15 but not FGF21. These results further emphasize that the ability of metformin to lower glucose and induce GDF15 is independent of changes in FGF21, which is primarily expressed in the liver (27), and, therefore, also that the accumulation in the GI tract (7–9) is crucial for these effects of metformin.

The strengths of our study include the randomized, cross-over design of the clinical study, which decreases the risk of confounding, and the examination of serum GDF15 and FGF21 in the glycogen-depleted fasting state in healthy individuals, which eliminated the influence of other factors known to regulate these stress-induced cytokines. Potential limitations of the present study include the lack of measurement of serum GDF15 and FGF21 before starting the 42 h fasting period to evaluate the effect of fasting alone and to exclude that the wash-out period was too short to remove the effect of metformin on GDF15 in those treated with the drug in the first period. Moreover, this is a secondary report, which means that the study was not designed to investigate the effect of metformin on GDF15 and FGF21.

In summary, our study demonstrates that the glucose-lowering effect of metformin, which was shown to be mediated by increased glycolysis, is accompanied by elevated circulating levels of the stress-induced cytokine GDF15 in healthy individuals after a 42 h fasting period. The lack of an increase in another stress-induced cytokine, FGF21, in the circulation, as well as the absent correlation between peak plasma metformin and the increase inserum GDF15 levels suggest that these actions of metformin are not mediated by its circulating concentrations. Consistent with our findings *in vivo*, metformin increased the transcription and secretion of GDF15 as well as reduced mitochondrial respiration and increased glycolysis in human intestinal Caco-2 cells. In contrast, transcription and secretion of FGF21 were barely detectable. Taken together, these findings corroborate with accumulating evidence that the glucose-lowering effect of metformin and its ability to induce and increase circulating GDF15 takes place in the intestine.

## Data Availability

The original contributions presented in the study are included in the article/**Supplementary Material**. Further inquiries can be directed to the corresponding author.

## Glossary

ATF4: Activating Transcription Factor 4
AUC: Area Under the Curve
DDIT3: DNA Damage Inducible Transcript 3
DMEM: Dulbecco’s Modified Eagle Medium
ECAR: Extracellular Acidification Rate
EGP: Endogenous Glucose Production
FBS: Fetal Bovine Serum
FCCP: Carbonyl cyanide-4 (trifluoromethoxy) phenylhydrazone
FFA: Free Fatty Acids
GDF15: Growth Differentiation Factor 15
GFRAL: Glial cell line-Derived Neurotrophic Factor Receptor Alpha-Like
GI: Gastrointestinal
HFD: High-fat diet
ISR: Integrated Stress Response
NEAA: Non-Essential Amino Acid
NOGM: Non-Oxidative Glucose Metabolism
OCR: Oxygen Consumption Rate
PBS: Phosphate Buffered Saline
qPCR: Quantitative PCR
Ra: Rate of appearance
Rd: Rate of disposal
REE: Resting Energy Expenditure
RER: Respiratory exchange ratio
