# Supplementary material for: Metformin increases glycolysis and the stress-induced cytokine GDF15 but not FGF21 in humans
Source: Front Endocrinol (Lausanne). 2026 Mar 18;17:1797525. doi: 10.3389/fendo.2026.1797525 (PMC13038576; doi:10.3389/fendo.2026.1797525)
Supplement: Supplementary file 1 [file DataSheet1.pdf]

Supplementary Table S1: Clinical and biochemical characteristics

Characteristics	
n (female/male)	34 (12/22)
Age (years)	27.3 ± 5.6
Weight (kg)	75.3 ± 11.9
Height (cm)	177.8 ± 10.3
BMI (kg/m^2)	23.7 ± 2.4
HbA1c (%)	5.1 ± 0.2
HbA1c (mmol/mol)	32.7 ± 2.4
HDL (mmol/l)	1.4 ± 0.3
LDL (mmol/l)	2.9 ± 0.7
Total cholesterol (mmol/l)	4.9 ± 0.8
Plasma triacylglycerol (mmol/l)	1.2 ± 0.9
eGFR (ml/min)	107.1 ± 19.2
Plasma creatinine (µmol/l)	77.4 ± 10.5

Baseline characteristics of the participants. BMI: body mass index, HDL: high-density lipoprotein, LDL: low-density lipoprotein, eGFR: estimated glomerular filtration rate. The data are presented as means ±SD.

Supplementary Table S2: Clinical effects of metformin on glucose and lipid metabolism

	**Placebo**	**Metformin**	**p-value**
Plasma glucose (mmol/l)	3.6 ± 0.1	3.6 ± 0.1	0.73
Serum insulin (pmol/l)	12 ± 1	14 ± 1	0.13
Serum C-peptide (pmol/l)	196 ± 10	198 ± 11	0.85
Plasma FFA (μmol/l)	1195 ± 42	1272 ± 62	0.15
Plasma lactate (mmol/l)	0.9 ± 0.0	1.0 ± 0.0	0.0015
Rd	55.2 ± 0.9	68.1 ± 1.1	8.9×10^{-13}
EGP	54.0 ± 1.0	67.2 ± 1.2	7.6×10^{-13}
NOGM	49.6 ± 3.7	64.4 ± 2.2	0.002
Glycolytic flux	43.9 ± 0.9	57.2 ± 1.1	1.7×10^{-11}
Glucose oxidation	6.0 ± 4.0	3.9 ± 2.2	0.61
Lipid oxidation	45.6 ± 2.5	48.1 ± 2.2	0.57
RER	0.74 ± 0.01	0.73 ± 0.00	0.14
REE (kcal/day)	1639 ± 59	1667 ± 51	0.62

Metabolic rates are expressed as mg mg^{-1} min^{-2}. Rd; rate of glucose disposal, EGP; endogenous glucose production, NOGM; non-oxidative glucose metabolism, RER; respiratory exchange ratio, REE; Resting energy expenditure. The data are presented as mean ±SEM.

Supplementary Table S3: Relationship between circulating levels of GDF15 and FGF21 and clinical, biochemical and metabolic characteristics

	r	p-value
	**Baseline serum GDF15**	
Height	-0.47	0.005
Weight	-0.50	0.003
BMI	-0.28	0.11
Age	-0.03	0.87
Plasma glucose	-0.48	0.004
Plasma FFA	0.05	0.76
Plasma Lactate	-0.01	0.96
EGP	-0.39	0.02
Rd	-0.33	0.056
Glycolytic flux	0.03	0.89
NOGM	0.01	0.94
Glucose oxidation	-0.07	0.69
Lipid oxidation	-0.07	0.71
REE	-0.29	0.09
	**Baseline serum FGF21**	
Height	-0.22	0.22
Weight	-0.28	0.10
BMI	0.19	0.29
Age	-0.07	0.69
Plasma glucose	-0.33	0.056
Plasma FFA	0.34	0.051
Plasma Lactate	-0.04	0.84
EGP	-0.07	0.70
Rd	-0.06	0.74
Glycolytic flux	0.34	0.049
NOGM	0.18	0.32
Glucose oxidation	-0.19	0.30
Lipid oxidation	0.31	0.09
REE	0.01	0.97
	**Δ serum GDF15**	
Plasma metformin (max)	-0.14	0.43
Plasma metformin (AUC)	-0.15	0.41
Δ Rd	-0.07	0.71
Δ EGP	-0.08	0.66
Δ Glycolytic flux	-0.04	0.84

Rd; rate of glucose disposal, EGP; endogenous glucose production, NOGM; non-oxidative glucose metabolism, REE; Resting energy expenditure, AUC; Area under curve.

Supplementary Fig. S1

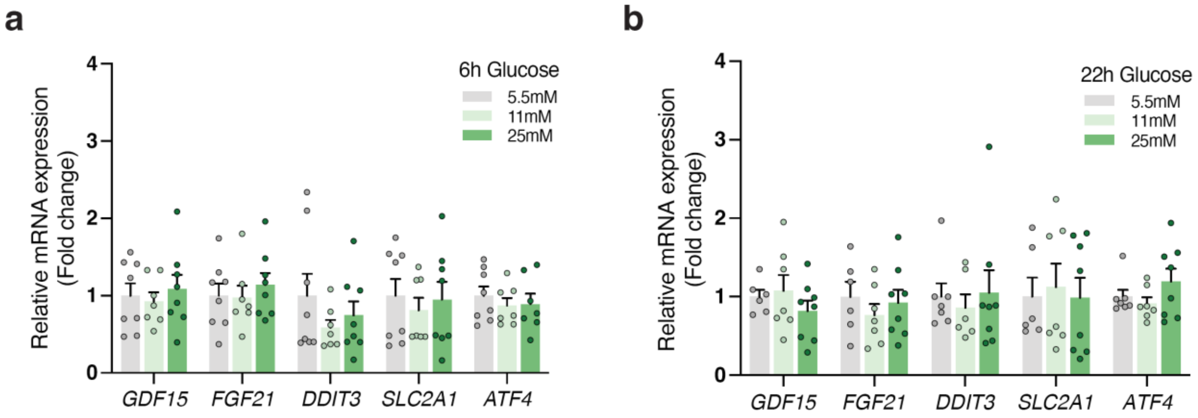

Fig. S1 Glucose does not affect the mRNA expression of selected genes. Relative mRNA expression of *GDF15*, *FDF21*, *DDIT3*, *SLC2A1* and *DDIT3* in differentiated Caco-2 cells **(a)** 6 h or **(b)** 22 h after media was changed from high glucose media to media with controlled glucose concentrations of either 5.5 mM, 11 mM or 25 mM. n = 6-8, 2 wells from 4 independent experiments evaluated in parallel. The data is presented as mean +/- SEM.

Supplementary Fig. S2

Supplementary Fig. S2

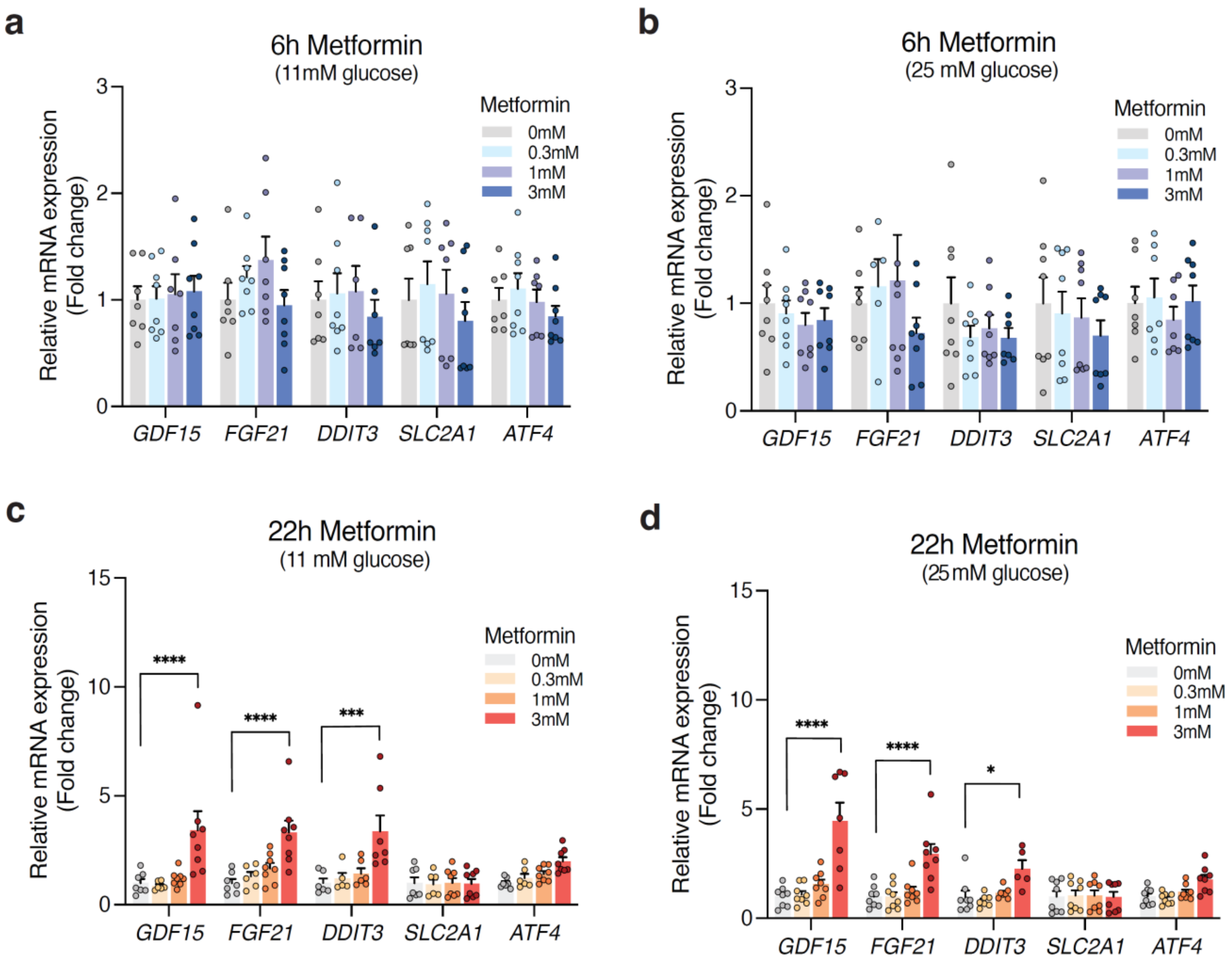

Fig. S2 Chronic metformin treatment increases mRNA levels of GDF15 and DDIT3 in Caco-2 cells. Transcript levels of *GDF15*, *FGF21*, *DITT3*, *SLC2A1*, and *ATF4* in differentiated Caco-2 cells following 6 h of metformin treatment maintained in media with 11 mM glucose **(a)** or 25 mM glucose **(b)**, or following 22 h of metformin treatment maintained in media with 11 mM glucose **(c)** or 25 mM **(d)**. The cells were either treated with a solution without metformin (0 mM) or with 0.3 mM, 1 mM, or 3 mM metformin. n = 6-8, 2 wells from 4 independent experiments evaluated in parallel. The data is presented as mean +/- SEM. *p<0.05, ***p<0.001, ****p<0.0001.

Supplementary Fig. S3

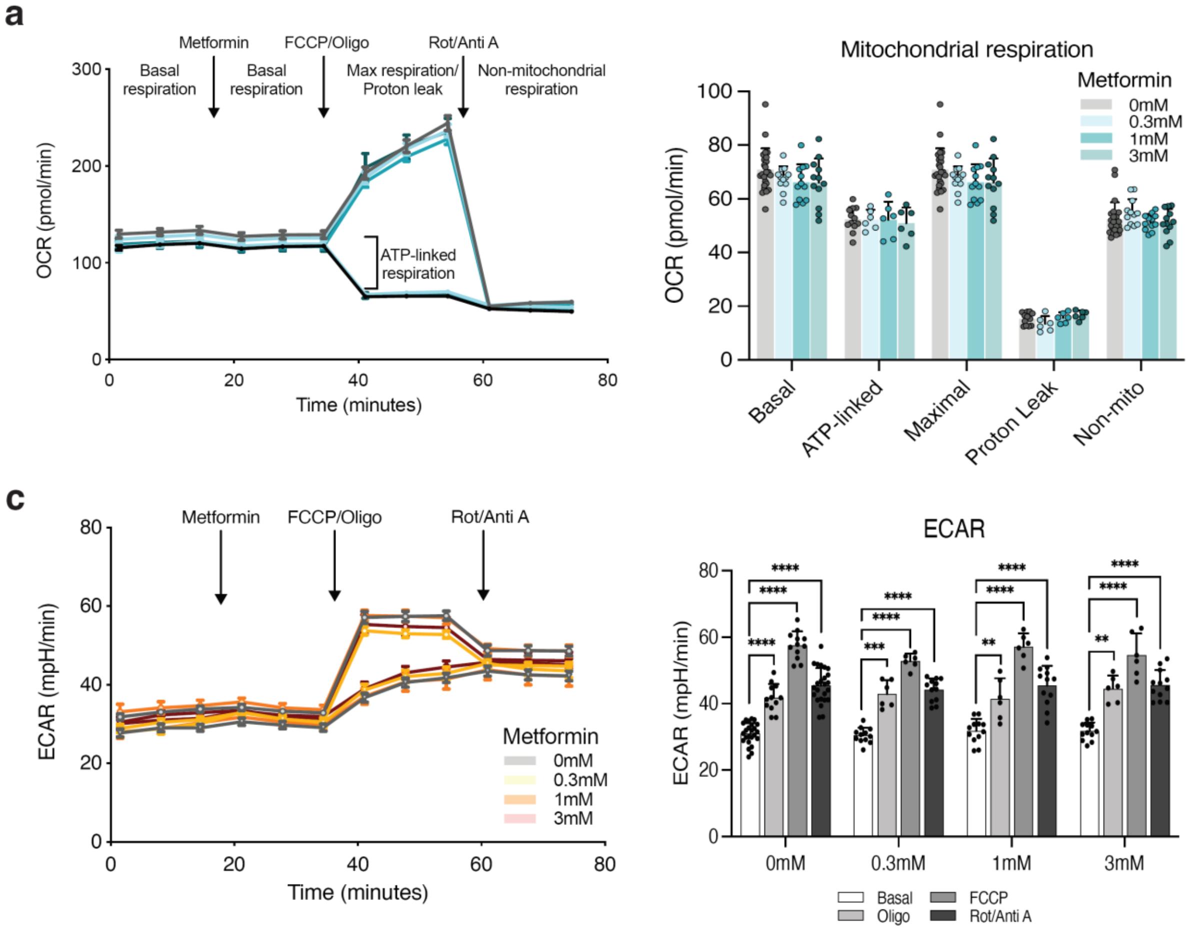

Fig. S3 Acute metformin treatment does not alter mitochondrial respiration or glycolytic acidification in intestinal cells. (a) Changes in oxygen consumption rate (OCR) and (b) extracellular acidification rate (ECAR) during a mitochondrial stress test of Caco-2 cells treated with acute doses of metformin at 0.3 mM, 1 mM or 3 mM or left untreated (0 mM). Arrows indicate the timepoint for when metformin and mitochondrial inhibitors were added. Oligo (Oligomycin) were added at 2pM, FCCP (Carbonyl cyanide-4 (trifluoromethoxy) phenylhydrazone) at 1pM, and Rot/Anti A (Rotenone, and Antimycin A) at 0.5pM. n=24 wells for baseline and Rot/Anti A measurements of OCR (pmol/min) and ECAR (mph/min) in controls. n=12 for OCR and ECAR at baseline or after Rot/Anti injection in metformin-treated wells (i.e., 0.3 mM, 1 mM, and 3 mM). Half of the plate was treated with either oligo or FCCP, (n=12 for vehicle and n=6 for metformin-treated cells). The data is presented as mean +/- SEM. **p<0.01, ****p<0.0001.
